# (*E*)-3-{[(2-Bromo-3-methyl­phen­yl)imino]­meth­yl}benzene-1,2-diol: crystal structure and Hirshfeld surface analysis

**DOI:** 10.1107/S2056989019015718

**Published:** 2019-11-26

**Authors:** Onur Erman Doğan, Necmi Dege, Erbil Ağar, Igor O. Fritsky

**Affiliations:** a Ondokuz Mayıs University, Faculty of Arts and Sciences, Department of Chemistry, 55139 Samsun, Turkey; b Ondokuz Mayıs University, Faculty of Arts and Sciences, Department of Physics, 55139 Samsun, Turkey; c Department of Chemistry, Taras Shevchenko National University of Kyiv, 64, Vladimirska Str., Kiev 01601, Ukraine

**Keywords:** crystal structure, Schiff base, O⋯O inter­action, Hirshfeld surface analysis, hydrogen bonds

## Abstract

The title compound, C_14_H_12_BrNO_2_, was synthesized by the condensation reaction of 2,3-di­hydroxy­benzaldehyde and 2-bromo-3-methyl­aniline. It crystallizes in the centrosymmetric triclinic space group *P* . The configuration about the C=N bond is *E*. The dihedral angle between the planes of the 5-(2-bromo-3-methyl­phenyl ring and the catechol ring is 2.80 (17)°. In the crystal, O—H⋯O hydrogen-bond inter­actions consolidate the crystal packing.

## Abstract 2

In the title Schiff base derivative carrying a 2-bromo-3-methyl­phenyl group, the conformation about the C=N bond is *E*. In the crystal, O—H⋯O hydrogen-bond inter­actions consolidate the crystal packing. A Hirshfeld surface analysis and fingerprint plots were used to further investigate the inter­molecular inter­actions in the solid state.

## Chemical context   

Schiff bases containing an azomethine or imine (–C=N–) unit are condensation products of primary amines and carbonyl compounds that were first reported by Hugo Schiff (1864 ▸). Schiff bases have a wide variety of applications in many areas of biological, organic and inorganic chemistry. The medicinal uses and applications of Schiff bases and their metal complexes are of increasing clinical and commercial importance and are increasingly significant in the medicinal and pharmaceutical fields because of their extensive range of biological activities (Karthikeyan *et al.*, 2006 ▸).
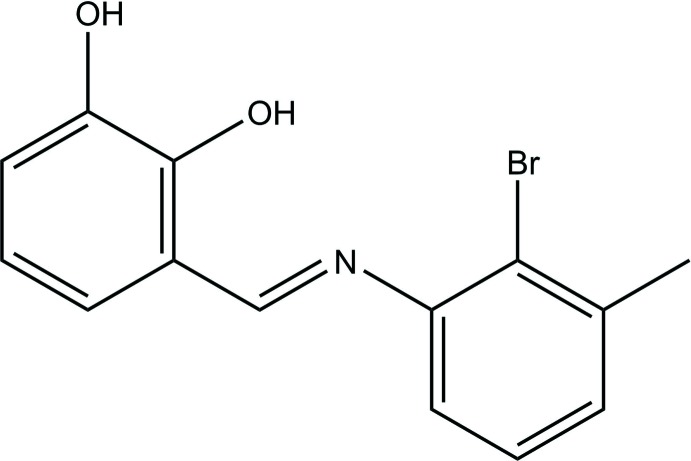


## Structural commentary   

**Figure 1:**
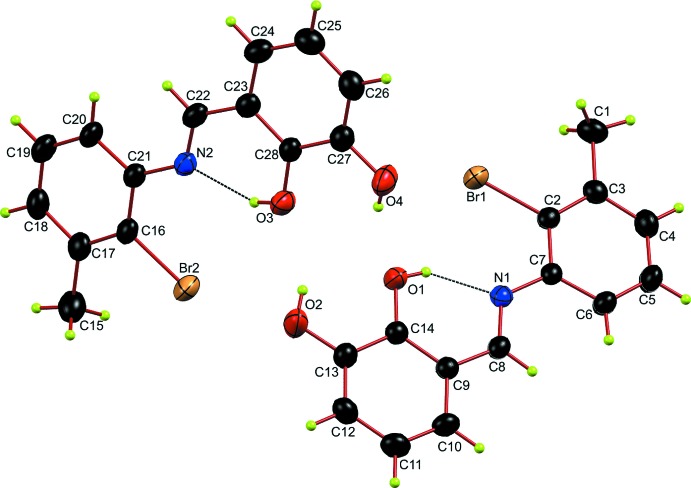
The mol­ecular structure of the title compound with the atomic numbering scheme. The dashed lines indicate the intra­molecular O—H⋯N hydrogen bonds. Displacement ellipsoids are drawn at the 30% probability level.

The structure of the title compound is shown in Fig. 1 ▸. It crystallizes in the centrosymmetric *P* 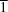 space group with *Z* = 4 (*Z*′ = 2). The two crystallographically independent mol­ecules have nearly the same geometrical parameters and the primary difference between them is the rotational orientation of H2 and H4*A*. The discussion will therefore be limited to that of the mol­ecule containing O1. The mol­ecular structure is constructed from two individually planar rings. The whole mol­ecule is approximately planar, with a maximum deviation of 0.117 (3) Å from planarity for the hydroxyl O1 atom of the catechol ring. The dihedral angle between the two benzene ring planes is 2.80 (17)°. The methyl C1 atom deviates from the plane of the C2–C7 benzene ring by 0.039 (2) Å while C9 deviates from the plane of the C9–C14 benzene ring by 0.024 (3) Å. The C8—N1—C7—C6 and C14— C9—C8—N1 torsion angles are −1.6 (5) and −1.1 (5)°, respectively. The planar mol­ecular conformation of each molecule is stabilized by an intra­molecular O—H⋯N hydrogen bond (Table 1 ▸).

## Supra­molecular features   

**Figure 2:**
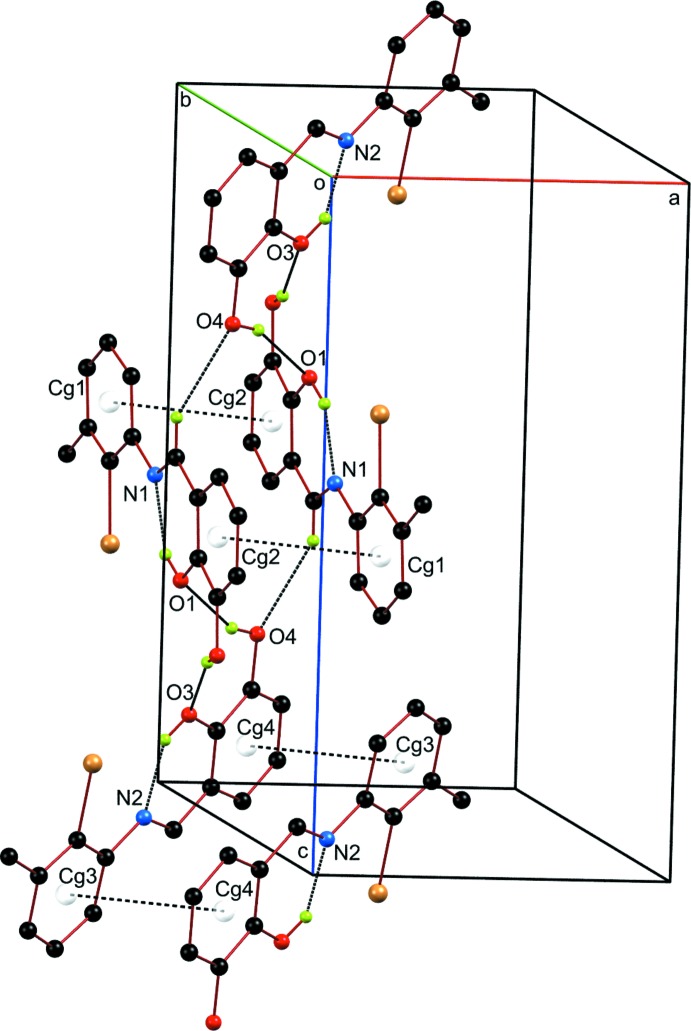
A partial view of the crystal packing of the title compound. Intra- and inter­molecular hydrogen bonds are shown as dotted lines while the π-stacking inter­actions are depicted by dashed lines.

In the crystal, the Schiff base units are linked by O—H⋯O and C—H⋯O hydrogen bonds (O4—H4*A*⋯O1, O2—H2⋯O3 and C8—H8⋯O4^i^; symmetry code as in Table 1 ▸), forming a tape structure along the *a-*axis direction (Fig. 2 ▸). The tapes are stacked into layers parallel to the benzene plane *via* π–π inter­actions (Fig. 2 ▸) with centroid–centroid distances of 3.750 (2) and 3.783 (2) Å, respectively, for *Cg*1⋯*Cg*2(1 − *x*, 1 − *y*, 1 − *z*) and *Cg*3⋯*Cg*4(−*x*, 1 − *y*, −*z*), where *Cg*1, *Cg*2, *Cg*3 and *Cg*4 are the centroids of the C2–C7, C9–C14, C16–C21 and C23–C28 rings, respectively.

## Database survey   

A search of the Cambridge Structural Database (CSD, version 5.40, update Nov 2018; Groom *et al.*, 2016 ▸) for the (*E*)-*N*-(2-bromo­phen­yl)-1-phenyl­methanimine skeleton yielded nine hits. The N1—C8 bond in the title structure is the same length within standard uncertainties as those in the structures of 2-bromo-*N*-salicylideneaniline (Burr & Hobson, 1969 ▸), *N*-(2-bromo­phen­yl)-1-(2-fluoro­phen­yl)methanimine (Kaur & Choudhury, 2014 ▸), 2-[(*E*)-(2,4-di­bromo­phenyl­imino)­meth­yl]-4-bromo­phenol (Bharti *et al.*, 2017 ▸), *N*-(2-bromo-4-methyl­phen­yl)naphthaldimine (Elmali *et al.*, 1998 ▸), *N*-(2-methyl­benzyl­idene)-2-bromo­aniline (Ojala *et al.*, 2007 ▸), 2-{[(2-bromo­phen­yl)imino]­meth­yl}-4-chloro­phenol (Guo, 2011 ▸), 2-{[(2-bromo­phen­yl)imino]­meth­yl}-4-chloro­phenol (Zhao & Zhang, 2012 ▸), 2-{[(2-bromo­phen­yl)imino]­meth­yl}-6-methyl­phenol (Karadağ *et al.*, 2010 ▸), 2-{[(2-bromo­phen­yl)imino]­meth­yl}-4-(tri­fluoro­meth­oxy)phenol (Tanak *et al.*, 2012 ▸). The C=N bond lengths in these structures vary from 1.270 (3) to 1.295 (5) Å and the C—O bond lengths from 1.336 (5) to 1.366 (2) Å. The mol­ecular conformations of these structures are also not planar, with dihedral angles between the phenyl rings varying between 5.00 (5) and 47.62 (9)°. It is likely that the intra­molecular O—H⋯N hydrogen bond, where the imine N atom acts as an hydrogen-bond acceptor, is an important prerequisite for the tautomeric shift toward the phenol–imine form. In fact, in all eight structures of the phenol–imine tautomers, hydrogen bonds of this type are observed.

## Hirshfeld surface analysis   

**Figure 3:**
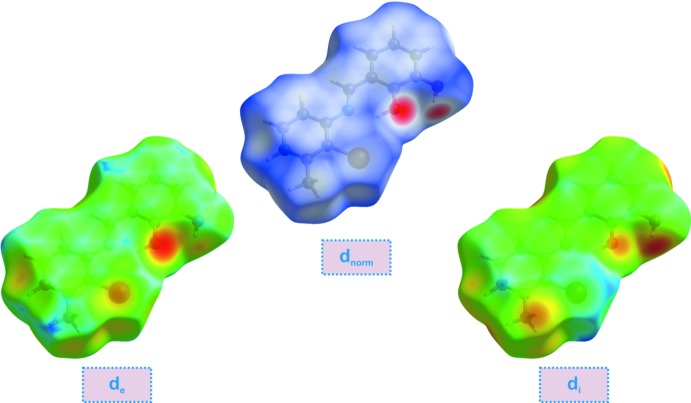
View of the three-dimensional Hirshfeld surface of the title compound plotted over *d*_norm_, *d*_e_ and *d*_i_.

**Figure 4:**
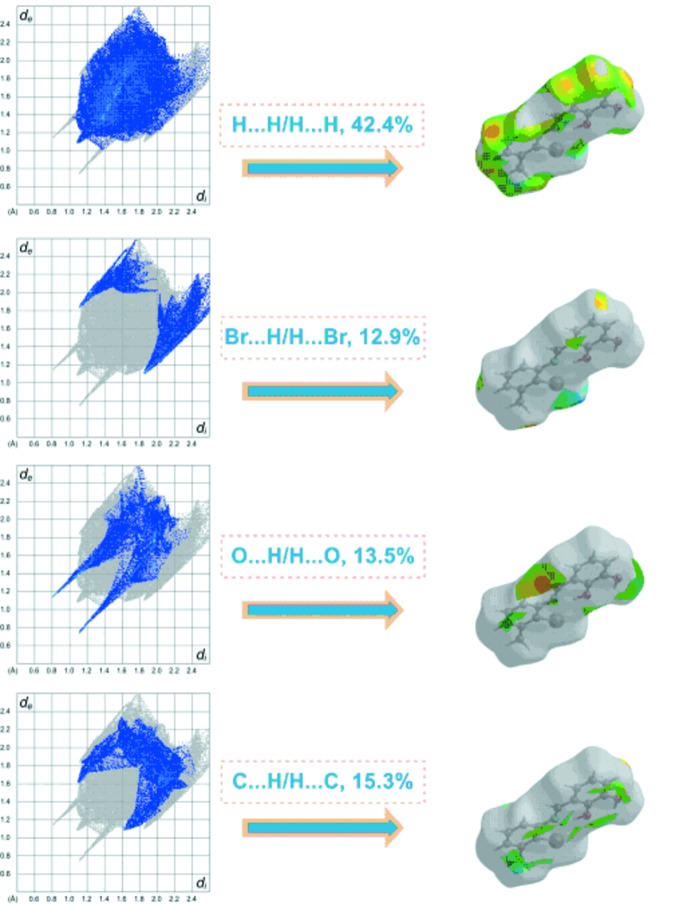
Two-dimensional fingerprint plots of the crystal with the relative contributions of the atom pairs to the Hirshfeld surface.

**Figure 5:**
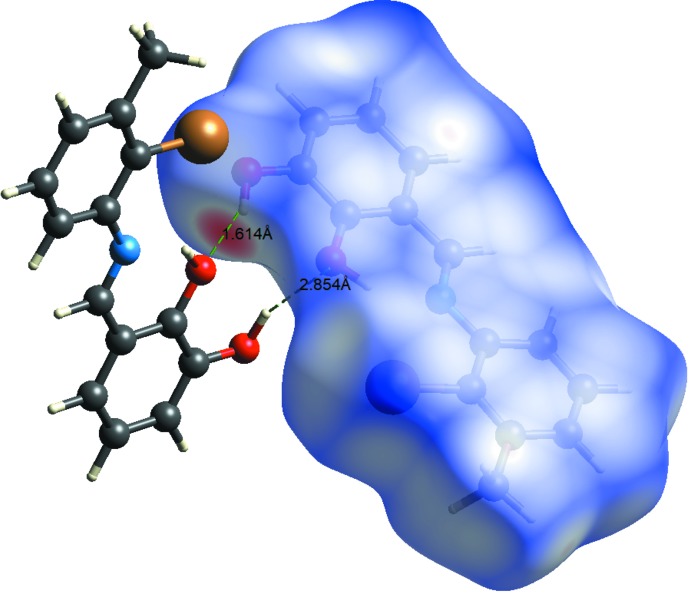
Hirshfeld surface mapped over *d*_norm_ to visualize the inter­molecular inter­actions.

Hirshfeld surface analysis of the title compound was performed utilizing the *CrystalExplorer* program (Turner *et al.*, 2017 ▸). The three-dimensional *d*_norm_ surface is a useful tool for analysing and visualizing the inter­molecular inter­actions and utilizes the function of the normalized distances *d*_e_ and *d*_i_, where *d*_e_ and *d*_i_ are the distances from a given point on the surface to the nearest atom outside and inside, respectively. The blue, white and red colour convention used for the *d*_norm_-mapped Hirshfeld surfaces indicates the inter­atomic contacts longer, equal to or shorter than the van der Waals separations. The standard-resolution mol­ecular three-dimensional (*d*_norm_) plot with *d*_e_ and *d*_i_ for the title compound is shown in Fig. 3 ▸. The bright-red spots near the oxygen and hydrogen atoms indicate donors and acceptors of a potential O—H⋯O inter­action. As can be seen from the two-dimensional fingerprint plots (scattering points spread up to *d*_e_ = *d*_i_ = 1.5 Å; Fig. 4 ▸), the dominant inter­action in the title compound originates from H⋯H contacts, which are the major contributor (42.4%) to the total Hirshfeld surface. The contribution from the O⋯H/H⋯O contacts (13.5%) is represented by a pair of sharp spikes that are characteristic of hydrogen-bonding inter­actions (Fig. 4 ▸). Other significant inter­actions are Br⋯H/H⋯Br (12.9%) and C⋯H/H⋯C (15.3%). While it is likely there are other identifiable points of contact that can be highlighted in the crystal, these may be of limited significance and do not require detailed discussion nor illustration. The inter­actions are visualized in Fig. 5 ▸.

## Synthesis and crystallization   

A mixture of 2,3-di­hydroxy­benzaldehyde (34.5 mg, 0.25 mmol) and 2-bromo-3-methyl­aniline (46.5 mg, 0.25 mmol) was stirred with ethanol (30 mL) at 377 K for 5 h, affording the title compound (49.73 mg, yield 65% m.p. 410–412 K). Single crystals suitable for X-ray measurements were obtained by recrystallization from ethanol at room temperature.

## Refinement   

Crystal data, data collection and structure refinement details are summarized in Table 2 ▸. The hy­droxy H atom was located in a difference-Fourier map, and the hy­droxy group was allowed to rotate during the refinement procedure (AFIX 147); O—H = 0.82 Å with *U*_iso_(H) = 1.5*U*_eq_(O). The C-bound H atoms were positioned geometrically and refined using a riding model: C—H = 0.93 Å with *U*_iso_(H) = 1.2*U*_eq_(C) for aromatic H atoms and C—H = 0.96 Å with *U*_iso_(H) = 1.5*U*_eq_(C) for methyl H atoms.

## Supplementary Material

Crystal structure: contains datablock(s) I. DOI: 10.1107/S2056989019015718/mw2151sup1.cif

Structure factors: contains datablock(s) I. DOI: 10.1107/S2056989019015718/mw2151Isup2.hkl

Supporting information file. DOI: 10.1107/S2056989019015718/mw2151Isup3.cml

CCDC reference: 1967023

Additional supporting information:  crystallographic information; 3D view; checkCIF report

## Figures and Tables

**Table 1:** Hydrogen-bond geometry (Å, °)

*D*—H⋯*A*	*D*—H	H⋯*A*	*D*⋯*A*	*D*—H⋯*A*
O1—H1⋯N1	0.82	1.85	2.571 (3)	146
O3—H3⋯N2	0.82	1.85	2.560 (3)	145
O4—H4*A*⋯O1	0.82	2.02	2.790 (4)	157
O2—H2⋯O3	0.82	2.11	2.875 (3)	156
C8—H8⋯O4^i^	0.93	2.54	3.383 (4)	151

Symmetry code: (i) 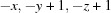.

**Table 2:** Experimental details

Crystal data	
Chemical formula	C_14_H_12_BrNO_2_
*M*_r_	306.16
Crystal system, space group	Triclinic, *P* 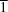
Temperature (K)	296
*a*, *b*, *c* (Å)	8.2301 (5), 10.1593 (6), 15.9428 (9)
α, β, γ (°)	102.496 (5), 90.597 (5), 103.213 (5)
*V* (Å^3^)	1264.46 (13)
*Z*	4
Radiation type	Mo *K*α
μ (mm^−1^)	3.24
Crystal size (mm)	0.49 × 0.31 × 0.21

Data collection	
Diffractometer	Stoe *IPDS* 2
Absorption correction	Integration (*X-RED32*; Stoe & Cie, 2002 ▸)
*T*_min_, *T*_max_	0.441, 0.663
No. of measured, independent and observed [*I* > 2σ(*I*)] reflections	13105, 4958, 3352
*R*_int_	0.044
(sin θ/λ)_max_ (Å^−1^)	0.617

Refinement	
*R*[*F* ^2^ > 2σ(*F* ^2^)], *wR*(*F* ^2^), *S*	0.038, 0.081, 0.97
No. of reflections	4958
No. of parameters	331
H-atom treatment	H-atom parameters constrained
Δρ_max_, Δρ_min_ (e Å^−3^)	0.38, −0.26

Computer programs: *X-AREA* and *X-RED32* (Stoe & Cie, 2002 ▸), *SHELXT2018* (Sheldrick, 2015*a* ▸), *SHELXL2018* (Sheldrick, 2015*b* ▸), *ORTEP-3 for Windows* and *WinGX* (Farrugia, 2012 ▸), *Mercury* (Macrae *et al.*, 2006 ▸) and *PLATON* (Spek, 2009 ▸).

## supplementary crystallographic information

### Crystal data

**Table:**

C_14_H_12_BrNO_2_	*Z* = 4
*M**_r_* = 306.16	*F*(000) = 616
Triclinic, *P*1	*D*_x_ = 1.608 Mg m^−^^3^
*a* = 8.2301 (5) Å	Mo *K*α radiation, λ = 0.71073 Å
*b* = 10.1593 (6) Å	Cell parameters from 15203 reflections
*c* = 15.9428 (9) Å	θ = 2.1–32.4°
α = 102.496 (5)°	µ = 3.24 mm^−^^1^
β = 90.597 (5)°	*T* = 296 K
γ = 103.213 (5)°	Column, red
*V* = 1264.46 (13) Å^3^	0.49 × 0.31 × 0.21 mm

### Data collection

**Table:**

Stoe IPDS 2 diffractometer	4958 independent reflections
Radiation source: sealed X-ray tube, 12 x 0.4 mm long-fine focus	3352 reflections with *I* > 2σ(*I*)
Plane graphite monochromator	*R*_int_ = 0.044
Detector resolution: 6.67 pixels mm^-1^	θ_max_ = 26.0°, θ_min_ = 2.1°
rotation method scans	*h* = −10→10
Absorption correction: integration (X-RED32; Stoe & Cie, 2002)	*k* = −12→12
*T*_min_ = 0.441, *T*_max_ = 0.663	*l* = −19→19
13105 measured reflections	

### Refinement

**Table:**

Refinement on *F*^2^	Primary atom site location: intrinsic phasing
Least-squares matrix: full	Hydrogen site location: inferred from neighbouring sites
*R*[*F*^2^ > 2σ(*F*^2^)] = 0.038	H-atom parameters constrained
*wR*(*F*^2^) = 0.081	*w* = 1/[σ^2^(*F*_o_^2^) + (0.0365*P*)^2^] where *P* = (*F*_o_^2^ + 2*F*_c_^2^)/3
*S* = 0.97	(Δ/σ)_max_ = 0.001
4958 reflections	Δρ_max_ = 0.38 e Å^−^^3^
331 parameters	Δρ_min_ = −0.26 e Å^−^^3^
0 restraints	

### Special details

**Table:**

Geometry. All esds (except the esd in the dihedral angle between two l.s. planes) are estimated using the full covariance matrix. The cell esds are taken into account individually in the estimation of esds in distances, angles and torsion angles; correlations between esds in cell parameters are only used when they are defined by crystal symmetry. An approximate (isotropic) treatment of cell esds is used for estimating esds involving l.s. planes.

### Fractional atomic coordinates and isotropic or equivalent isotropic displacement parameters (Å^2^)

**Table:**

	*x*	*y*	*z*	*U*_iso_*/*U*_eq_	
Br1	0.47553 (5)	0.73422 (3)	0.43378 (2)	0.06496 (13)	
Br2	0.27549 (6)	0.20843 (4)	0.05124 (2)	0.07628 (15)	
O1	0.1481 (3)	0.4451 (2)	0.34610 (15)	0.0653 (7)	
H1	0.211056	0.487560	0.388310	0.098*	
O3	0.1364 (4)	0.4958 (2)	0.16060 (16)	0.0691 (7)	
H3	0.189385	0.466512	0.120398	0.104*	
N1	0.2564 (3)	0.5099 (2)	0.50502 (16)	0.0470 (6)	
N2	0.2469 (3)	0.4778 (3)	0.01003 (16)	0.0532 (7)	
O4	0.0206 (4)	0.6518 (3)	0.29811 (17)	0.0822 (8)	
H4A	0.064074	0.586678	0.297637	0.123*	
O2	−0.0441 (4)	0.2540 (3)	0.21486 (16)	0.0897 (9)	
H2	0.016348	0.330320	0.214834	0.135*	
C7	0.3645 (4)	0.6053 (3)	0.57103 (19)	0.0457 (7)	
C9	0.0451 (4)	0.3067 (3)	0.4473 (2)	0.0469 (7)	
C8	0.1507 (4)	0.4002 (3)	0.5163 (2)	0.0490 (7)	
H8	0.143775	0.382141	0.571109	0.059*	
C2	0.4759 (4)	0.7195 (3)	0.5508 (2)	0.0458 (7)	
C14	0.0503 (4)	0.3320 (3)	0.3637 (2)	0.0500 (8)	
C16	0.3400 (4)	0.2699 (3)	−0.0509 (2)	0.0531 (8)	
C21	0.3160 (4)	0.3988 (3)	−0.0583 (2)	0.0525 (8)	
C23	0.1469 (4)	0.6720 (3)	0.0803 (2)	0.0538 (8)	
C22	0.2142 (4)	0.5943 (4)	0.0084 (2)	0.0584 (9)	
H22	0.234551	0.629736	−0.040566	0.070*	
C28	0.1130 (4)	0.6197 (3)	0.1544 (2)	0.0525 (8)	
C12	−0.1530 (5)	0.1178 (3)	0.3120 (2)	0.0645 (9)	
H12	−0.218773	0.053589	0.266809	0.077*	
C3	0.5838 (4)	0.8201 (3)	0.6109 (2)	0.0534 (8)	
C17	0.4073 (4)	0.1854 (4)	−0.1138 (2)	0.0613 (9)	
C26	0.0233 (5)	0.8244 (4)	0.2207 (3)	0.0676 (10)	
H26	−0.017827	0.875467	0.267836	0.081*	
C27	0.0531 (4)	0.6990 (4)	0.2243 (2)	0.0603 (9)	
C13	−0.0486 (4)	0.2341 (3)	0.2961 (2)	0.0586 (9)	
C10	−0.0661 (4)	0.1862 (3)	0.4616 (2)	0.0593 (9)	
H10	−0.073250	0.169793	0.516793	0.071*	
C20	0.3657 (5)	0.4421 (4)	−0.1328 (2)	0.0656 (10)	
H20	0.351806	0.527272	−0.140140	0.079*	
C11	−0.1625 (5)	0.0941 (4)	0.3947 (3)	0.0669 (10)	
H11	−0.235219	0.014899	0.404432	0.080*	
C4	0.5836 (5)	0.8024 (4)	0.6942 (2)	0.0641 (9)	
H4	0.656723	0.867053	0.736383	0.077*	
C24	0.1153 (5)	0.8017 (4)	0.0781 (3)	0.0666 (10)	
H24	0.136398	0.837047	0.029072	0.080*	
C6	0.3679 (5)	0.5952 (3)	0.6565 (2)	0.0597 (9)	
H6	0.294647	0.522244	0.673039	0.072*	
C5	0.4778 (5)	0.6914 (4)	0.7163 (2)	0.0710 (11)	
H5	0.481124	0.681646	0.772972	0.085*	
C18	0.4550 (5)	0.2344 (4)	−0.1862 (2)	0.0675 (10)	
H18	0.501793	0.180660	−0.229667	0.081*	
C25	0.0539 (5)	0.8763 (4)	0.1473 (3)	0.0710 (10)	
H25	0.032795	0.961647	0.145094	0.085*	
C19	0.4346 (5)	0.3615 (4)	−0.1952 (2)	0.0743 (11)	
H19	0.468248	0.392695	−0.244387	0.089*	
C1	0.6961 (5)	0.9453 (3)	0.5889 (3)	0.0729 (11)	
H1B	0.774499	0.916334	0.549065	0.109*	
H1C	0.755569	1.004995	0.640409	0.109*	
H1D	0.629921	0.994500	0.563320	0.109*	
C15	0.4311 (6)	0.0463 (4)	−0.1053 (3)	0.0857 (12)	
H15A	0.501966	0.057822	−0.054737	0.129*	
H15B	0.482042	0.005655	−0.155138	0.129*	
H15C	0.324472	−0.013470	−0.100577	0.129*	

### Atomic displacement parameters (Å^2^)

**Table:**

	*U*^11^	*U*^22^	*U*^33^	*U*^12^	*U*^13^	*U*^23^
Br1	0.0871 (3)	0.0575 (2)	0.0514 (2)	0.01015 (18)	0.01253 (18)	0.02160 (16)
Br2	0.1074 (3)	0.0777 (3)	0.0517 (2)	0.0241 (2)	0.0085 (2)	0.02912 (19)
O1	0.0832 (18)	0.0563 (13)	0.0492 (13)	−0.0038 (12)	−0.0037 (12)	0.0181 (11)
O3	0.095 (2)	0.0653 (14)	0.0560 (15)	0.0260 (13)	0.0267 (13)	0.0235 (12)
N1	0.0517 (16)	0.0429 (13)	0.0485 (15)	0.0138 (12)	0.0063 (12)	0.0120 (11)
N2	0.0559 (17)	0.0566 (15)	0.0434 (15)	0.0037 (13)	0.0064 (12)	0.0133 (12)
O4	0.110 (2)	0.096 (2)	0.0532 (15)	0.0456 (17)	0.0244 (15)	0.0214 (14)
O2	0.115 (2)	0.0826 (18)	0.0498 (15)	−0.0168 (16)	−0.0007 (14)	0.0115 (13)
C7	0.0526 (19)	0.0452 (15)	0.0429 (16)	0.0179 (14)	0.0054 (14)	0.0107 (13)
C9	0.0467 (19)	0.0469 (16)	0.0495 (18)	0.0145 (14)	0.0074 (14)	0.0123 (14)
C8	0.052 (2)	0.0548 (17)	0.0459 (17)	0.0204 (15)	0.0081 (15)	0.0160 (14)
C2	0.0482 (19)	0.0482 (15)	0.0479 (17)	0.0212 (14)	0.0097 (14)	0.0144 (13)
C14	0.052 (2)	0.0486 (16)	0.0497 (18)	0.0118 (14)	0.0095 (15)	0.0107 (14)
C16	0.050 (2)	0.0652 (19)	0.0398 (17)	0.0051 (16)	−0.0037 (14)	0.0126 (15)
C21	0.050 (2)	0.0598 (18)	0.0447 (18)	0.0037 (15)	0.0022 (15)	0.0155 (15)
C23	0.0451 (19)	0.0603 (18)	0.0529 (19)	0.0031 (15)	0.0017 (15)	0.0158 (15)
C22	0.053 (2)	0.068 (2)	0.054 (2)	0.0024 (17)	0.0030 (16)	0.0263 (17)
C28	0.051 (2)	0.0578 (18)	0.0454 (18)	0.0059 (15)	0.0039 (15)	0.0117 (15)
C12	0.062 (2)	0.0557 (19)	0.066 (2)	0.0038 (17)	0.0031 (18)	0.0029 (16)
C3	0.052 (2)	0.0503 (17)	0.059 (2)	0.0159 (15)	0.0051 (16)	0.0100 (15)
C17	0.058 (2)	0.072 (2)	0.050 (2)	0.0108 (18)	−0.0099 (17)	0.0095 (17)
C26	0.059 (2)	0.072 (2)	0.067 (2)	0.0160 (19)	0.0002 (18)	0.0036 (18)
C27	0.057 (2)	0.066 (2)	0.054 (2)	0.0095 (17)	0.0022 (16)	0.0113 (16)
C13	0.063 (2)	0.0601 (19)	0.051 (2)	0.0121 (17)	0.0086 (16)	0.0112 (16)
C10	0.056 (2)	0.0600 (19)	0.065 (2)	0.0098 (17)	0.0154 (17)	0.0250 (17)
C20	0.082 (3)	0.070 (2)	0.0445 (19)	0.0119 (19)	0.0161 (18)	0.0191 (17)
C11	0.057 (2)	0.059 (2)	0.081 (3)	0.0024 (17)	0.012 (2)	0.0190 (19)
C4	0.062 (2)	0.067 (2)	0.057 (2)	0.0120 (18)	−0.0103 (17)	0.0047 (17)
C24	0.065 (2)	0.065 (2)	0.074 (3)	0.0099 (18)	0.0035 (19)	0.0310 (19)
C6	0.067 (2)	0.0626 (19)	0.0497 (19)	0.0089 (17)	0.0052 (17)	0.0206 (16)
C5	0.085 (3)	0.079 (2)	0.046 (2)	0.008 (2)	−0.0057 (19)	0.0187 (18)
C18	0.065 (2)	0.086 (3)	0.046 (2)	0.016 (2)	0.0061 (18)	0.0049 (18)
C25	0.067 (3)	0.059 (2)	0.086 (3)	0.0149 (19)	0.003 (2)	0.014 (2)
C19	0.084 (3)	0.089 (3)	0.052 (2)	0.013 (2)	0.017 (2)	0.026 (2)
C1	0.068 (3)	0.056 (2)	0.089 (3)	0.0034 (18)	0.000 (2)	0.0165 (19)
C15	0.106 (4)	0.092 (3)	0.069 (3)	0.045 (3)	0.002 (2)	0.015 (2)

### Geometric parameters (Å, º)

**Table:**

Br1—C2	1.903 (3)	C12—C11	1.391 (5)
Br2—C16	1.905 (3)	C12—H12	0.9300
O1—C14	1.330 (3)	C3—C4	1.379 (5)
O1—H1	0.8200	C3—C1	1.502 (4)
O3—C28	1.340 (4)	C17—C18	1.379 (5)
O3—H3	0.8200	C17—C15	1.504 (5)
N1—C8	1.295 (4)	C26—C27	1.364 (5)
N1—C7	1.408 (4)	C26—C25	1.388 (5)
N2—C22	1.277 (4)	C26—H26	0.9300
N2—C21	1.413 (4)	C10—C11	1.361 (5)
O4—C27	1.370 (4)	C10—H10	0.9300
O4—H4A	0.8200	C20—C19	1.361 (5)
O2—C13	1.354 (4)	C20—H20	0.9300
O2—H2	0.8200	C11—H11	0.9300
C7—C6	1.390 (4)	C4—C5	1.373 (5)
C7—C2	1.404 (4)	C4—H4	0.9300
C9—C14	1.411 (4)	C24—C25	1.368 (5)
C9—C10	1.415 (4)	C24—H24	0.9300
C9—C8	1.421 (4)	C6—C5	1.363 (5)
C8—H8	0.9300	C6—H6	0.9300
C2—C3	1.377 (5)	C5—H5	0.9300
C14—C13	1.399 (5)	C18—C19	1.377 (5)
C16—C17	1.381 (5)	C18—H18	0.9300
C16—C21	1.397 (5)	C25—H25	0.9300
C21—C20	1.388 (4)	C19—H19	0.9300
C23—C28	1.403 (4)	C1—H1B	0.9600
C23—C24	1.408 (5)	C1—H1C	0.9600
C23—C22	1.436 (5)	C1—H1D	0.9600
C22—H22	0.9300	C15—H15A	0.9600
C28—C27	1.392 (5)	C15—H15B	0.9600
C12—C13	1.367 (4)	C15—H15C	0.9600
			
C14—O1—H1	109.5	C26—C27—O4	118.4 (3)
C28—O3—H3	109.5	C26—C27—C28	121.0 (3)
C8—N1—C7	124.1 (3)	O4—C27—C28	120.7 (3)
C22—N2—C21	123.9 (3)	O2—C13—C12	119.2 (3)
C27—O4—H4A	109.5	O2—C13—C14	120.7 (3)
C13—O2—H2	109.5	C12—C13—C14	120.1 (3)
C6—C7—C2	116.9 (3)	C11—C10—C9	120.1 (3)
C6—C7—N1	124.1 (3)	C11—C10—H10	120.0
C2—C7—N1	119.0 (3)	C9—C10—H10	120.0
C14—C9—C10	119.1 (3)	C19—C20—C21	120.8 (4)
C14—C9—C8	120.6 (3)	C19—C20—H20	119.6
C10—C9—C8	120.2 (3)	C21—C20—H20	119.6
N1—C8—C9	121.8 (3)	C10—C11—C12	120.4 (3)
N1—C8—H8	119.1	C10—C11—H11	119.8
C9—C8—H8	119.1	C12—C11—H11	119.8
C3—C2—C7	123.4 (3)	C5—C4—C3	121.4 (3)
C3—C2—Br1	119.0 (2)	C5—C4—H4	119.3
C7—C2—Br1	117.6 (2)	C3—C4—H4	119.3
O1—C14—C13	118.4 (3)	C25—C24—C23	120.7 (3)
O1—C14—C9	122.3 (3)	C25—C24—H24	119.7
C13—C14—C9	119.3 (3)	C23—C24—H24	119.7
C17—C16—C21	123.3 (3)	C5—C6—C7	120.4 (3)
C17—C16—Br2	118.8 (3)	C5—C6—H6	119.8
C21—C16—Br2	118.0 (3)	C7—C6—H6	119.8
C20—C21—C16	116.9 (3)	C6—C5—C4	121.0 (3)
C20—C21—N2	124.1 (3)	C6—C5—H5	119.5
C16—C21—N2	118.9 (3)	C4—C5—H5	119.5
C28—C23—C24	118.9 (3)	C19—C18—C17	121.1 (4)
C28—C23—C22	120.2 (3)	C19—C18—H18	119.4
C24—C23—C22	121.0 (3)	C17—C18—H18	119.4
N2—C22—C23	121.8 (3)	C24—C25—C26	119.9 (4)
N2—C22—H22	119.1	C24—C25—H25	120.1
C23—C22—H22	119.1	C26—C25—H25	120.1
O3—C28—C27	118.5 (3)	C20—C19—C18	120.7 (3)
O3—C28—C23	122.3 (3)	C20—C19—H19	119.6
C27—C28—C23	119.1 (3)	C18—C19—H19	119.6
C13—C12—C11	121.0 (3)	C3—C1—H1B	109.5
C13—C12—H12	119.5	C3—C1—H1C	109.5
C11—C12—H12	119.5	H1B—C1—H1C	109.5
C2—C3—C4	116.8 (3)	C3—C1—H1D	109.5
C2—C3—C1	122.8 (3)	H1B—C1—H1D	109.5
C4—C3—C1	120.4 (3)	H1C—C1—H1D	109.5
C18—C17—C16	117.1 (3)	C17—C15—H15A	109.5
C18—C17—C15	120.2 (4)	C17—C15—H15B	109.5
C16—C17—C15	122.7 (3)	H15A—C15—H15B	109.5
C27—C26—C25	120.5 (4)	C17—C15—H15C	109.5
C27—C26—H26	119.8	H15A—C15—H15C	109.5
C25—C26—H26	119.8	H15B—C15—H15C	109.5
			
C8—N1—C7—C6	−1.6 (5)	Br2—C16—C17—C15	0.3 (5)
C8—N1—C7—C2	179.0 (3)	C25—C26—C27—O4	−179.9 (3)
C7—N1—C8—C9	−179.6 (3)	C25—C26—C27—C28	−0.7 (5)
C14—C9—C8—N1	−1.1 (5)	O3—C28—C27—C26	−178.5 (3)
C10—C9—C8—N1	178.5 (3)	C23—C28—C27—C26	1.6 (5)
C6—C7—C2—C3	−0.9 (5)	O3—C28—C27—O4	0.8 (5)
N1—C7—C2—C3	178.5 (3)	C23—C28—C27—O4	−179.2 (3)
C6—C7—C2—Br1	179.2 (2)	C11—C12—C13—O2	−179.4 (4)
N1—C7—C2—Br1	−1.4 (4)	C11—C12—C13—C14	−0.4 (6)
C10—C9—C14—O1	178.0 (3)	O1—C14—C13—O2	0.2 (5)
C8—C9—C14—O1	−2.4 (5)	C9—C14—C13—O2	−178.7 (3)
C10—C9—C14—C13	−3.1 (5)	O1—C14—C13—C12	−178.8 (3)
C8—C9—C14—C13	176.5 (3)	C9—C14—C13—C12	2.2 (5)
C17—C16—C21—C20	−1.0 (5)	C14—C9—C10—C11	2.0 (5)
Br2—C16—C21—C20	179.3 (2)	C8—C9—C10—C11	−177.5 (3)
C17—C16—C21—N2	−179.7 (3)	C16—C21—C20—C19	0.0 (5)
Br2—C16—C21—N2	0.6 (4)	N2—C21—C20—C19	178.6 (3)
C22—N2—C21—C20	4.1 (5)	C9—C10—C11—C12	−0.2 (6)
C22—N2—C21—C16	−177.3 (3)	C13—C12—C11—C10	−0.7 (6)
C21—N2—C22—C23	−178.8 (3)	C2—C3—C4—C5	−1.5 (6)
C28—C23—C22—N2	−0.9 (5)	C1—C3—C4—C5	177.7 (4)
C24—C23—C22—N2	178.5 (3)	C28—C23—C24—C25	0.6 (5)
C24—C23—C28—O3	178.5 (3)	C22—C23—C24—C25	−178.9 (3)
C22—C23—C28—O3	−2.1 (5)	C2—C7—C6—C5	−1.3 (5)
C24—C23—C28—C27	−1.5 (5)	N1—C7—C6—C5	179.3 (3)
C22—C23—C28—C27	177.9 (3)	C7—C6—C5—C4	2.0 (6)
C7—C2—C3—C4	2.3 (5)	C3—C4—C5—C6	−0.6 (6)
Br1—C2—C3—C4	−177.8 (3)	C16—C17—C18—C19	−0.6 (5)
C7—C2—C3—C1	−176.9 (3)	C15—C17—C18—C19	−179.9 (4)
Br1—C2—C3—C1	3.0 (5)	C23—C24—C25—C26	0.4 (6)
C21—C16—C17—C18	1.3 (5)	C27—C26—C25—C24	−0.4 (6)
Br2—C16—C17—C18	−179.0 (2)	C21—C20—C19—C18	0.7 (6)
C21—C16—C17—C15	−179.5 (3)	C17—C18—C19—C20	−0.3 (6)

### Hydrogen-bond geometry (Å, º)

**Table:**

*D*—H···*A*	*D*—H	H···*A*	*D*···*A*	*D*—H···*A*
O1—H1···N1	0.82	1.85	2.571 (3)	146
O3—H3···Br2	0.82	2.86	3.499 (2)	136
O3—H3···N2	0.82	1.85	2.560 (3)	145
O4—H4*A*···O1	0.82	2.02	2.790 (4)	157
O4—H4*A*···O3	0.82	2.32	2.731 (4)	112
O2—H2···O1	0.82	2.29	2.724 (3)	114
O2—H2···O3	0.82	2.11	2.875 (3)	156
C8—H8···O4^i^	0.93	2.54	3.383 (4)	151

Symmetry code: (i) −*x*, −*y*+1, −*z*+1.
